# Differential persistence of neutralizing antibody against SARS-CoV-2 in post immunized Bangladeshi population

**DOI:** 10.1038/s41598-022-18302-9

**Published:** 2022-08-29

**Authors:** Dipa Roy, Md. Mosfequr Rahman, Arnaba Saha Chaity, Md. Abu Reza, Ariful Haque

**Affiliations:** 1 Molecular Pathology Laboratory, Institute of Biological Sciences, University of Rajshahi, Rajshahi, 6205 Bangladesh; 2 Department of Population Science and Human Resource Development, University of Rajshahi, Rajshahi, 6205 Bangladesh; 3 Molecular Biology and Protein Science Laboratory, Department of Genetic Engineering and Biotechnology, University of Rajshahi, Rajshahi, 6205 Bangladesh

**Keywords:** Immunology, Microbiology

## Abstract

Development of effective vaccines have been immensely welcomed by the world to prevent the transmission of SARS-CoV-2. However, the duration and clinical implications of antibody-mediated natural immunity in SARS-CoV-2 have not been adequately elucidated alongside some other immune system transforming factors. In a cohort study, we measured NAb titer following the 2nd immunization dosage of the CoviShield (AZD1222) vaccine*.* The enzyme-linked immunoassay was used to look for SARS-CoV-2—specific NAb. We measured NAb at 30 days after the 2nd dosage of immunization and > 96% titer was detected in 42.9% of subjects, but only 5.1% of subjects retained the same level after 180 days. The median NAb titer dropped significantly, from 92% at 30 days to 58% at 180 days (p < 0.001). Besides, there were significant differences observed in NAb titer after 180 days by age, sex, COVID-19 infection, tobacco use, and asthma patients. However, SARS-CoV-2 infection along with two dosages of immunization upheld NAb titer (*p* < 0.001) even at the end of the study period.

## Introduction

Severe Acute Respiratory Syndrome Corona virus 2 (SARS-CoV-2) has continued to show a devastating impact on the global population particularly on the immunosuppressed and people with co-morbidity^1–3^. This catastrophic circumstance has been prevalent until effective immunization were introduced. In this process, virus-specific neutralizing antibody (NAb) is a very important factor for reducing viral replication and increasing viral clearance^4,5^. Neutralizing antibodies primarily target the SARS-CoV-2 S protein's receptor-binding domain (RBD)^6–8^, thereby inhibiting viral entrance into the cell. In the case of SARS CoV-2 infection^9^ or immunization^10^, neutralizing antibodies emerge quickly in the human circulatory system and lasts for few months^11,12^. Though this doesn’t promise ultimate protection as several variants of concern (VOCs) have evolved by mutating the S gene of the virus.

The CoviShield COVID-19 (AZD1222) (C19VAZ) vaccine, formerly known as ChAdOx1 nCoV-19, is made from a weakened version of a common cold virus (ChAdOx1), where the genetic material has been added to produce SARS-CoV-2 Spike glycoprotein^13^. According to the World Health Organization (WHO), the AZD1222 vaccine has been shown to be 63.09% effective against symptomatic SARS-CoV-2 infection, which is correlated to longer dosage intervals within the 8–12 weeks range for better vaccine efficacy. The Bangladesh government has initiated mass-level vaccination since early February 2021 using AZD1222. Though the mass level of vaccination is ongoing, the transmission of the disease is still in an oscillating phase. Nonetheless, protective immunity after immunization is currently unclear as the immune responses are fading away and new viral variants are spreading at the same time^14^. This raises the dilemma of designing a vaccine that has induced selection pressure for the formation of a viral variation as well as immune response convalescence^15^. Although memory B cells and T cells both play a part in the fight against SARS-CoV-2, there is good evidence that NAb play a major role since passive immunization can lower the severity of the disease^16^. As a result, we have concentrated our research on demonstrating in vitro viral neutralization titer in CoviShield vaccinated persons in Bangladesh in a cohort setting. In addition, we compared the NAb response between 30 and 180 days after immunization, concentrating on age, sex, some lifestyle factors (tobacco use and steroidal medications) and co-morbidities.

## Methods

### Study population characteristics

Serial blood samples were collected from 531 healthy adults (above 35 years of age) after 30 days and 180 days of completing the 2nd dosage of immunization. The samples were taken from physicians, nurses, and other employees of Rajshahi Medical College Hospital, Rajshahi, Bangladesh as a group who are more prone to SARS-CoV-2 exposure and the faculty staff of the University of Rajshahi, assumed to be less prone to SARS-CoV-2 exposure. The enzyme-linked immunoassay (ELISA) was used to look for SARS-CoV-2-specific NAb responses in the samples. A more detailed description of the subject’s cohort is given in Table 1. Sequential blood samples were collected from each person after completing immunization on May 15 and October 17, 2021 to measure and characterize the dynamic changes in virus-specific NAb titer. We considered as Covid positive samples for the study only those who were infected 30 days prior to vaccination. Any subject with Covid infection in between the study period (May 15 and October 17, 2021) was removed.

**Table 1:** Demographics and cohort characteristics of the study population (n = 531).

Characteristics	n (%)	Characteristics	n (%)
**Age, year**		**Tobacco user**	99 (18.6)
Mean (SD)	42.3 (10.5)	**Comorbidities**	
Median (IQR)	42 (35–50)	Type 2 Diabetes	63 (11.8)
**Age category, (n, %)**		Hypertension	132 (24.9)
< 50 years	390 (73.4)	Asthma	45 (8.5)
≥ 50 years	141 (26.6)	**NAb titers after 30 days of 2nd dosage**	
**Sex (n, %)**		Mean (SD)	83.4 (21.4)
Male	309(58.2)	Median (IQR)	92 (78–97)
Female	222 (41.8)	**NAb titer after 30 days of 2nd dosage (n, %)**	
**BMI**		< 70	90 (17.0)
Mean (SD)	26.8 (3.4)	70–95	213 (40.1)
Median (IQR)	25.6 (24.1–27.3)	≥ 96	228 (42.9)
**BMI cut-off (n, %)**		**NAb titers after 180 days of 2nd dosage**	
< 30	484 (91.1)	Mean (SD)	54.9 (27.0)
≥ 30	47 (8.9)	Median (IQR)	58 (32–77)
COVID-19 positive*	129 (24.3)	**NAb titer after 180 days of 2nd dosage (n, %)**	
**Profession**		< 70	318 (59.9)
Medical staff	298 (56.1)	70–95	186 (35.0)
Non-medical staff	233 (43.9)	≥ 96	27 (5.1)

*SD* standard deviation, *IQR* interquartile range, *NAb* neutralizing antibody.

*Subjects who tested positive 30 days prior vaccination.

### Sample collection

All subjects had venous blood samples taken in the amount of 5 ml in Vacutainer tubes after completing the consent form. The serum was collected and tested after spinning the blood at 4000 rpm for 10 min at room temperature. The serum samples were then aliquoted and stored at − 80 °C until the tests were performed.

### Ethical approval

Approval of this study protocol and ethical clearance was obtained from the Institutional Animal, Medical Ethics, Biosafety, and Biosecurity Committee (IAMEBBC) for Experimentations on Animal, Human, Microbes and Living Natural Sources, under supervision of Institute of Biological Sciences (IBSc), University of Rajshahi, Rajshahi, Bangladesh (Memo number: 58/320/IAMEBBC/IBSc). Further this was followed in accordance with guidelines of IAMEBBC and as per the protocol all the participants provided written informed consent.

### SARS-CoV-2 specific neutralizing antibody detection in plasma/serum

The COVID-19 Neutralizing Antibody Microlisa test kits supplied by J. Mitra & Co. Pvt. Ltd., New Delhi, India, were used to test SARS-CoV-2 specific NAb in serum samples according to the manufacturer's recommendations (Lot No. ECN020521). The assay kit was targeted to detect neutralizing antibodies generated against SARS-CoV-2 in human serum/plasma in an in vitro semi-quantitative manner, preventing the interaction between the viral Spike glycoprotein's receptor binding domain (RBD) and the cell surface receptor angiotensin converting enzyme-2 (ACE2). The detection protocols and routines adhered to the manufacturer’s instructions (https://jmitra.co.in/wp-content/uploads/2021/10/Instruction-Manual-Covid-19-Neutralizing-Antibodies-Microlisa.pdf) are as follows:

To begin, 20 μl of sample/control and 180 μl of sample diluent buffer (included in the kit) was taken in a clean 1.5 ml micro-centrifuge tube (diluted serum sample, negative control and positive control at a volume ratio of 1:9 with sample diluent). 60 μl of working conjugate solution (Horseradish peroxidase conjugated recombinant SARS-CoV-2 receptor binding domain was diluted 1:50 in conjugate diluent buffer) was added to 120 μl of diluted sample/negative and positive control solution. Contents of each tubes were thoroughly mixed and incubated for 30 min at room temperature. 150 μl neutralized sample/control were added to microtiter wells coated with recombinant hACE2 protein. The plate was sealed using an adhesive plate sealer and Incubated at 37 °C for 30 min. The wells were rinsed five times with the working wash buffer solution (20 ml. of 25X wash buffer concentrate was mixed with 480 ml. of distilled or deionized water) after incubation to remove the unbound HRP-RBD-neutralizing antibody complex. Finally, each well received 150 μl of working substrate solution (TMB substrate and TMB Diluent was mixed in 1:1 ratio to prepare working substrate) containing chromogenic and hydrogen peroxide, which was further incubated for 15 min in the dark at room temperature (20–30 °C). A stop solution (1 N sulfuric acid) was used to bring the blue-colored reaction to a halt. A total of 100 μl of stop solution was taken into each well. Optical density (O.D.) was measured at 450 nm wavelength using Microtiter plate reader (AccuSkan FC, Fisher Scientific, Waltham, MA, USA).

The test evaluation was carried out following the recommended positive and negative cutoffs, and test results were interpreted by calculating inhibition rates for samples as follows:

$$\% \,{\text{ Inhibition}}\, = \,\left( {{1} - {\text{ Sample}}\,{\text{O}}.{\text{D}}./{\text{Negative}}\,{\text{Control }}\,{\text{O}}.{\text{D}}.} \right)\, \times \,{1}00\%$$

According to the manufacturer’s instructions, neutralizing antibody levels higher than 30%, were considered as positive (SARS-CoV-2 Neutralizing Antibody present).

Besides the negative cutoff control provided by the manufacturer, serum sample from non-immunized and confirmed non-infected subjects were also used to ratify the results.

### Statistical analysis

Descriptive statistics have been presented as frequencies and percentages for categorical variables and as means (standard deviation, SD) and medians (interquartile range, IQR) for continuous variables. Besides assessing continuous NAb titer after 30 and 180 days of immunization, we also categorized NAb titer into three groups: < 70, 70–95 and ≥ 96. To compare the variables for categorical NAb titer (< 70, 70–95, ≥ 96) and continuous NAb titer values, a chi-square test and a Student's t-test were used. A Wilcoxon signed rank test was used to evaluate the difference in NAb titer values after 30 days and 180 days of the 2nd dosage of immunization. Additionally, an unpaired, two-tailed t-test with Welch’s correction was used for comparing Nab titers between different socio-demographic and health-related variables. The association between Nab titer after 30 and 180 days of the 2nd dosage and potential factors, i.e., age, sex, BMI, profession, tobacco use, COVID-19 positive (30 days before vaccination), and co-morbidities, was estimated by generalized linear models. The level of significance for this set of analyses was set at a 2-tailed *p* < 0.05, with 95% CIs. All statistical analyses were performed with the use of STATA 16 MP (Stata Corp., College Station, TX).

## Results

### Trial population

Information on the 531 subjects such as demographic data is shown in Table 1, the subjects had a median age of 42 years (IQR, 35–50), with 42% (222) being female and 27% (141) being 50 years old and having received the 2nd dosage of immunization. More than half of the subjects (56%) were medical professionals. Among the total subjects, 18.6% used tobacco, and 8.5% were on mild steroid therapy due to various clinical complications. Furthermore, diabetes, hypertension, and asthma were reported by around 12%, 25%, and 9% of the subjects, respectively (Table 1).

Antiviral humoral immunity, which is infrequently apparent more than a year after hospitalization, wanes over time, according to studies of MERS and SARS-CoV beta corona virus infections, two viruses closely related to SARS-CoV-2^17,18^. It is unknown whether SARS-CoV-2 NAb decline at the same pace as SARS-CoV NAb. However, the durability of protective immunity is unknown at this time, primary immune responses are inevitably waning^11,14,19^ and there is continual propagation of increasingly dangerous virus variations that may elude both vaccine-induced and convalescent immune responses^19^. Only a few studies have looked into the path of neutralization titers after two  dosage of immunization^20^.

**Figure 1:**
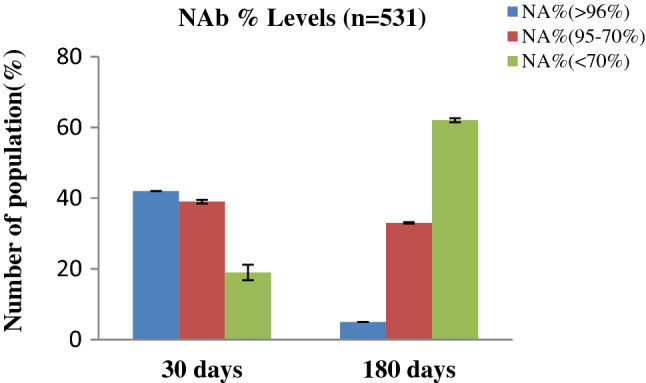
Percentage (%) of people with immune response on the basis of days, after 30 days and 180 days completing 2nd immunization.

NAb levels were first assessed immediately after the second immunization dosage. After 30 days, 42.9% of subjects had NAb levels greater than 96%, but only 5.1% had the same level after 180 days. At the same time, for the vast majority of people, the level had dropped significantly to 70% (See Fig. 1). The median Nab titer levels dropped considerably after the 2nd dosage of immunization, from 92 (IQR 78–97) at 30 days to 58 (IQR 32–77) at 180 days (*p* = 0.001). After 30 days, 228 (42.9%) of the 531 subjects had 96% NAb titer, 213 (40.1%) had 70–95% NAb titer, and 90 (17%) had 70% NAb titer. However, after 180 days, 27 (5.1%) subjects had ≥ 96%, 186 (35.0%) had 70–95%, and 318 (59.9%) had < 70% NAb titer (Table1).

**Figure 2:**
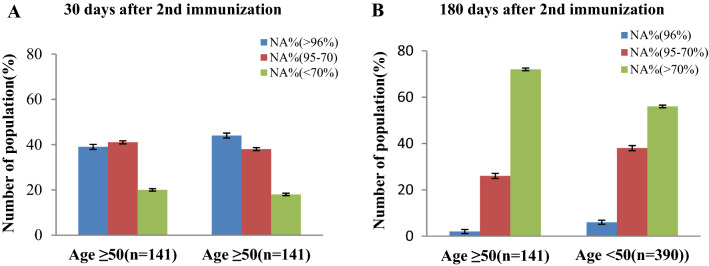
Percentage (%) of people with an immune response based on age group after 30 days (**A**) and 180 days (**B**) from 2nd immunization completion.

We discovered that individuals in the older age groups had a faster rate of NAb titer decline than those in the younger age groups. Similarly, 180 days after immunization, the NAb titer in the same group decreased (Fig. 2), which was alarming. NAb titer decreased at a higher rate in the older age groups than in the younger age groups (*p* = 0.001).

**Figure 3:**
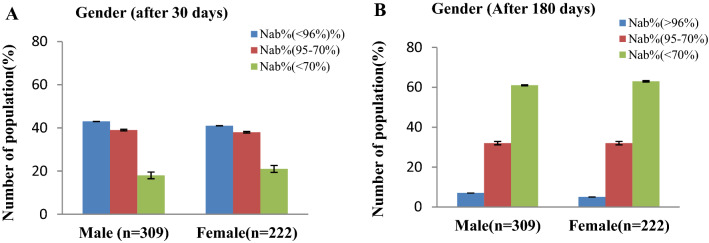
Percentage (%) of people with immune responses based on sex after 30 days (**A**) and 180 days (**B**) of 2nd immunization completion.

Gender discrepancy in COVID-19 disease severity shows higher mortality rate in male than female^21,22^, though Investigated Gender's impact on immunological memory shows that males have higher Spikes in IgG, nucleocapsid, and RBD IgG than females^11,23^. However, we discovered that the female had a slightly lower response to NAb production after immunization although not significant (*p* = 0.088) (Fig. 3). We presume that this is an outcome of technical error during vaccination because the female Bangladeshi wore tight clothing where access to the deltoid muscle was harder for vaccine providers. Personal communication with the vaccine providers also confirmed this observation. This is why some females had fewer NAb than after 30 days, though the waning ratio of NAb in females remained similar to males after 180 days.

**Figure 4:**
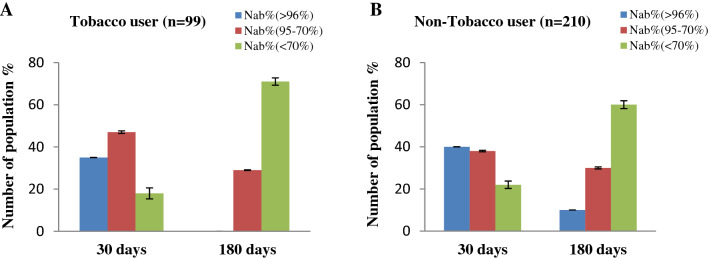
Percentage (%) people with immune response regarding (**A**) Tobacco user and (**B**) non-Tobacco user group (only considered male in our country’s context, females barely smoke in Bangladesh n = 309) after 30 days and 180 days of completing 2nd immunization.

**Figure 5:**
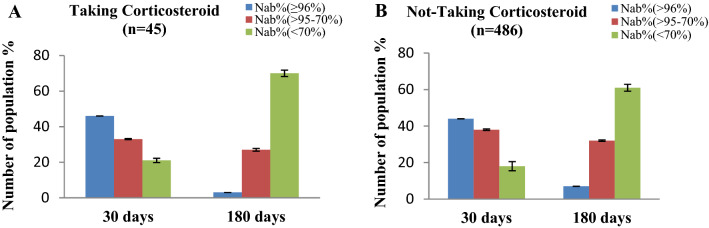
Percentage (%) of people with immune responses in (**A**) the Corticosteroid-taking group and (**B**) the non-Corticosteroid-taking group after 30 days and 180 days of completing 2nd immunization.

We further divided our study group on the basis of tobacco use (in the form of smoking, we only considered male in our country's context) and individuals on corticosteroids in inhaler form for complications like asthma and COPD. When compared to their non-smoking peers, vaccinated people who smoked or used corticosteroids have a lower NAb titer after immunization (Figs. 4 and 5). Furthermore, their NAb titer had fallen significantly (*p* < 0.001) after 180 days of immunization. The effects of corticosteroids were expected as they have an immune suppressant effect. We assume that the smoker group may have similar immune suppression due to their long-time smoking habit^24^.

**Figure 6:**
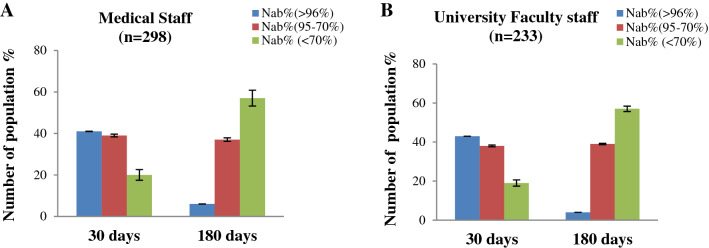
(**A**) After 30 days and (**B**) after 180 days of 2nd vaccination, the percentage of participants with an immune response in the Medical Staff and University Faculty Staff group.

In a mass immunization initiative, both front-line and academic staff were targeted for vaccination at the same time. This immunization program was in effect when the country was under lockdown and SARS-CoV-2 transmission was at its peak. In our study group, some of the medical professionals were survivors of the SARS-CoV-2 infection. However, medical professionals working in the hospital during the lockdown were more likely to be exposed to the virus than the university faculty members who were staying at home. Though the medical professionals were vaccinated twice, some of the doctors and nurses reported mild or subclinical infection of SARS-CoV-2 after immunization. Whereas, a very few University Faculty member reported the infection either pre or post immunization. Once we compared NAb titter between these two group medical professionals has a greater Nab responses than the university faculty group among the sampled subjects (Fig. 6). This is potentially because of the natural infection with SARS-CoV-2.

**Figure 7:**
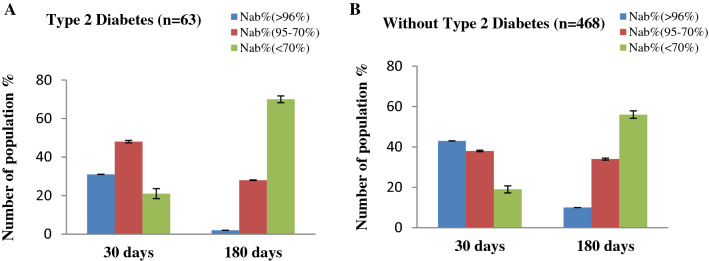
Percentage (%) of people with immune responses in (**A**) Type 2 Diabetes (**B**) Without Type 2 Diabetes after 30 days and 180 days of completing 2nd immunization.

**Figure 8:**
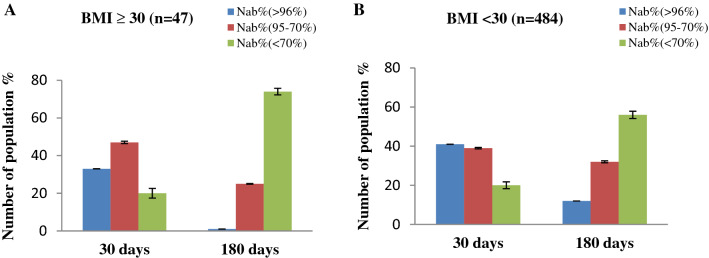
Percentage (%) of people with immune responses in (**A**) BMI ≥ 30 (**B**) BMI < 30 after 30 days and 180 days of completing 2nd immunization.

We further divided our subjects on the basis of type 2 Diabetes and BMI. Considering the type 2 Diabetes, the immunized people with type 2 Diabetes, shows a lower NAb titer after immunization (Fig. 7). Among subjects having type-2 Diabetes, NAb titers significantly (*p* < 0.001) decreased with a higher rate than subjects without Type-2 Diabetes. Again when considering the BMI, those who have BMI ≥ 30, have lower NAb titer after immunization (Fig. 8). We observed that the subjects having BMI ≥ 30 had significantly (*p* = 0.001) lower response to NAb production after than subjects having BMI < 30 after 180 days of immunization.

Table 2 presents the mean differences of NAb titer values after 30 and 180 days of completing the 2nd dosage of immunization by different categorical variables. We observed a statistically significant difference of mean NAb titer values after 30 days between BMI < 30 and ≥ 30 BMI (82.7 vs. 90.0; *p* = 0.012), COVID-19 negative and positive (81.7 vs. 88.9; *p* = 0.001), medical staffs and non-medical staffs (85.8 vs. 81.5; *p* = 0.022), tobacco users and non-users (78.6 vs. 84.5; *p* = 0.014), and having diabetes and not-having diabetes (89.5 vs. 82.6; *p* = 0.016). However, the statistically significant differences in mean Nab titer values after 180 days were observed between age < 50 years and ≥ 50 years (57.3 vs. 48.4; *p* = 0.001), COVID-19 negative and positive (52.3 vs. 63.1; *p* < 0.001), tobacco users and non-users (44.2 vs. 57.4; *p* < 0.001), suffering from hypertension and not (50.0 vs. 56.5; *p* = 0.015), and having asthma and not having asthma (37.4 vs. 59.1; *p* < 0.001).

**Table 2:** Bivariate associations between demographic and health-related variables, and NAb titers after 30 and 180 days of vaccination.

Characteristics	NAb titers after 30 days			NAb titers after180 days		
	Mean (SD)	*p*-value (t-test)	*p*-value ($\chi^{2}$-test)*	Mean (SD)	*p*-value (t-test)	*p*-value ($\chi^{2}$-test)*
**Age**		0.068	0.166		0.001	0.013
< 50 years	84.4 (20.6)			57.3 (26.2)		
≥ 50 years	80.6 (23.3)			48.4 (28.3)		
**Sex**		0.481	0.591		0.355	0.002
Male	84.0 (21.9)			55.8 (28.9)		
Female	82.6 (20.8)			53.6 (24.1)		
**BMI**		0.012	0.005		0.735	0.886
< 30	82.7 (21.2)			54.8 (26.9)		
≥ 30	90.0 (8.1)			56.2 (28.4)		
**COVID-19 positive**		0.001	< 0.001		< 0.001	< 0.001
No	81.7 (22.3)			52.3 (26.8)		
Yes	88.9 (17.6)			63.1 (26.2)		
**Profession**		0.022	0.004		0.488	0.525
Medical staff	85.8 (20.1)			54.2 (27.5)		
University faculty staff	81.5 (22.3)			55.8 (26.4)		
**Tobacco user**		0.014	0.936		< 0.001	0.012
No	84.5 (19.5)			57.4 (25.9)		
Yes	78.6 (27.9)			44.2 (29.3)		
**Comorbidities**						
Type 2 Diabetes		0.016	0.019		0.215	0.120
No	82.6 (22.3)			55.4 (27.4)		
Yes	89.5 (12.2)			51.0 (23.4)		
Hypertension		0.336	0.003		0.015	0.203
No	82.9 (21.7)			56.5 (26.7)		
Yes	85.0 (20.7)			50.0 (27.4)		
Asthma		0.695	0.415		< 0.001	0.045
No	83.6 (21.0)			59.1 (24.5)		
Yes	82.6 (23.5)			37.4 (30.1)		

*SD* standard deviation, *Nab* neutralizing antibody.

*p-values for chi-square test were calculated from the differentials in Nab titer categories (< 70, 70–95, ≥ 96) by different socio demographics and health-related variables included in Table 2.

### Factors associated with Nab titers after 30 and 180 days of the 2nd dosage of immunization

Table 3 presents the results from the multivariable analyses of the association between Nab titer values after 30 and 180 days of the 2nd dosage and potential factors. NAb titer after 30 days of vaccination were found to be negatively associated with age ≥ 50 years (β-coefficient:− 6.63, 95% CI [− 11.04 to− 2.22]; *p* = 0.003), medical staff (− 4.42 [− 8.02 to − 0.81]; *p* = 0.016), and tobacco users (− 8.48 [− 13.49 to − 3.47]; *p* = 0.001). However, for subjects with BMI** ≥ **30 (10.63 [4.34 to 16.90]; *p* = 0.001), COVID-19 positive (6.11 [1.94 to 10.29]; *p* = 004), and suffering from type 2 Diabetes (10.18 [4.16 to 16.21]; *p* = 0.001), there was a noteworthy positive association with NAb titer after 30 days of immunization. Results also show that NAb titer after 180 days significantly decreased among subjects aged 50 years and older (− 5.61 [− 10.87 to − 0.36]; *p* = 0.036), females (−6.39 [− 11.11 to − 1.68]; *p* = 0.008), tobacco users (− 14.56 [− 20.55 to − 8.58]; *p* < 0.001), and subjects suffering from asthma (− 19.93 [− 24.77 to − 14.00]; *p* < 0.001). However, a notable increase of NAb titer (9.08 [4.10 to 14.06]; *p* < 0.001) was observed among COVID-19 positive subjects after 180 days of vaccination. We also performed another set of multivariable analysis keeping the COVID-19 positive and negative subjects in separate groups. We found similar significance in NAbs titer within the variables even if we keep the infected and uninfected subjects in separate groups (Supplementary Tables 1, 2).

**Table 3:** Multivariable analyses of NAb titer after 30 days and 180 days of 2nd dosage vaccination.

Characteristics	NAb titers after 30 days		NAb titers after 180 days	
	β (95% CI)	*p*-value	β (95% CI)	*p*-value
**Age**				
< 50 years	Na		Na	
≥ 50 years	− 6.63 (− 11.04 to − 2.22)	0.003	− 5.61 (− 10.87 to − 0.36)	0.036
**Sex**				
Male	Na		Na	
Female	− 2.37 (− 6.31 to 1.58)	0.240	− 6.39 (− 11.11 to − 1.68)	0.008
**BMI ≥ 30**	10.63 (4.34 to 16.90)	0.001	5.01 (− 2.48 to 12.51)	0.190
**COVID-19 positive**	6.11 (1.94 to 10.29)	0.004	9.08 (4.10 to 14.06)	< 0.001
**Profession**				
Non-medical staff	Na		Na	
Medical staff	− 4.42 (− 8.02 to − 0.81)	0.016	− 1.58 (− 5.88 to 2.72)	0.472
**Tobacco user**	− 8.48 (− 13.49 to − 3.47)	0.001	− 14.56 (− 20.55 to − 8.58)	< 0.001
**Comorbidities**				
Type 2 Diabetes	10.18 (4.16 to 16.21)	0.001	− 2.25 (− 9.44 to 4.95)	0.540
Hypertension	1.72 (− 2.49 to 5.93)	0.422	− 4.23 (− 9.30 to 0.76)	0.096
Asthma	0.72 (− 3.79 to 5.23)	0.755	− 19.93 (− 24.77 to − 14.00)	< 0.001

*Nab* neutralizing antibody, *CI* confidence interval.

## Discussion

Virus-specific NAb have long been thought to be a key factor in viral clearance but this has been challenged as there is a continual propagation of increasingly dangerous variations of SARS-CoV-2 strains that can evade both vaccine-induced and convalescent immune responses^25^. The S glycoprotein's receptor-binding domain (RBD) and N-terminal domain (NTD) of SARS-CoV-2 detect and bind to the angiotensin-converting enzyme-2 (ACE-2) receptor, which is prerequisite for virus attachment and entry into host cells. The RBD, precisely the receptor-binding motif (RBM) region, also contains the main antigenic epitopes recognized by neutralizing antibodies (NAbs)^26,27^. Thus, any alteration in the S protein's structure can affect viral infection processes, providing the virus a selective advantage^28^. Therefore the Spike protein mutations are being extensively studied for their effects on disease transmission, pathophysiology, and vaccination efficacy as well^29^. However, until now, most of SARS-CoV-2 vaccines used for mass vaccination are designed to aim the S protein of the virus in order to generate NAbs against the RBM sections that ultimately inhibit viral binding sites to the ACE-2 receptor in host cells, and prevent infection^30^. Due to evolution process the virus have mutated several times, some of the changes in the Spike protein have an increased transmissibility^31,32^. This may be explained by an enhanced Spike-protein-binding affinity for the ACE2 receptor. For example, Alpha and Beta have been shown to have a 1.98 × and 4.62 × greater binding affinity than that of original strain^33^. Thus the efficacy of these vaccines were put to test once new variants comprised of a number of lineages and sub lineages (Delta B.1.617.2, Delta Plus AY.4.2, Mu B.1.621, Omicron BA.1, BA.1.1 and BA.2 etc.) started to transmit with new S protein mutations with the ability to evade host immunity or induced immune response^34–37^. The efficacy of CoviShield (AZD1222) against Alpha variant was promising and well performing against Delta variant to some extent^38,39^. This neutralization effect of vaccine was further reduced for variants in other lineages and sub-lineages^40^. For example, the Omicron variants had the ability to evade immunity because of its higher affinity for human ACE2 than Delta variants, indicating a higher transmission^41^. Due to the changes, antibodies established against prior lineages of SARS-CoV-2 are less effective in variants such as Omicron and Delta^42^. The causes driving this "antigenic shift" are likely to become stronger when the majority of the population develops resistance to the virus through infection, vaccination, or both^43^.

The SARS CoV-2 neutralizing antibody developed after immunization and retained in the blood for few months depending on several physiological factors^44^. Lower neutralization titer and reduced vaccine efficacy against distinct viral types have also been documented, in addition to the effect of declining neutralization titers with time^30,45–48^. As a result, we emphasized on in vitro viral neutralization titer found in vaccinated individual in a cohort manner.

We revealed that after two dosage of immunization, the NAbs titer decreased over time in relation to age, Type 2 Diabetes and their lifestyles (including body-mass index, use of tobacco, and medication like corticosteroids for asthma and COPD patients). Our findings show that 30 days after receiving the 2nd dosage of AZD1222, adults' NAb titer against SARS-CoV-2 are enhanced differentially. Conversely, in adults, after 180 days, there was a considerable drop in NAb titer. This decreasing trend is most noticeable in people over the age of 50 and smokers who have type-2 diabetes and high BMI. In case of the diabetic patients, a low-grade metabolic inflammation is seen that is similar to chronic inflammation found in obese individuals. This inflammation may weaken macrophage activation and blunt the mechanism of cytokine production^49,50^. Besides the obese and type 2 diabetic people have more compromised B cell and T cell response^51,52^. Patients with T2DM also have a poor humoral immune response, making them more susceptible to re-infection^53^. After two dosage of the Pfizer-BioNTech BNT162b2 mRNA vaccine, the T2DM group showed significantly lower antibody titers than the non-diabetic group^44^. These partially explains the vaccine escape mechanism and rapid reduction of NAbs in people with type 2 diabetes and high BMI. Our findings show that NAb titer in patients were varied, and that the protective humoral immune response to SARS-CoV-2 may fade over time, similar to what has been observed in patients infected with other human coronaviruses such as HCoV-229E^54,55^. Patients infected with COVID-19 have a short-term humoral immune response that is very similar to that seen in patients infected with SARS-CoV and MERS-CoV^56,57^, who have a rapid drop in virus-specific antibody titers within 3–4 months.

We also found that the NAb titer decreases over time as well as depends on the age group and their lifestyles (including use of tobacco, and medication like corticosteroids for asthma and COPD patients). In case of smokers’, we found that 30 days after receiving the 2nd dosage of AZD1222, NAb titer against SARS-CoV-2 are enhanced differentially. There is a diminishing trend noticeable in smokers. Smoking has been shown to raise the expression of ACE2^58^, the SARS-CoV-2 virus's receptor for cellular entry, and to increase the risk of severe COVID-19 illness in young adults^59^. Although omicron infection is 40–70% less severe in young people than Delta infections irrespective of ACE2 expression^60^.

Neutralizing antibody has not been linked to a reduction in COVID-19 disease severity and lacks the response of cell mediated immunity^15,61–64^, as has been found in the case of Middle Eastern respiratory syndrome (MERS), which is caused by infection with the human coronavirus MERS-CoV^63^. However, in nonhuman primates, neutralizing antibody is linked to protective immunity against secondary (2°) infection with SARS-CoV-2 or SARS-CoV^15,45–48^.Yet, the SARS CoV-2 neutralizing antibody is retained in the blood for several months. Lower neutralization titer and reduced vaccine efficacy against distinct viral types have also been documented, in addition to the effect of declining neutralization titers with time^30,65–68^.

Viral evolution is a continual process that, in the long term, might improve "viral fitness" and selective adaptation. As the variants of concern (VOCs) can be caused by new mutations in the S gene thus vaccine efficacy must be monitored on a continuous basis. If vaccines do not provide comprehensive protection against VOC variations, as with the H1N1 vaccine, periodic vaccine updates or reconstruction will be required. Novel vaccinations that generate NAbs against diverse variants by targeting highly conserved antigenic epitopes of the S protein are further options.

## Supplementary Information

Supplementary Tables.

## Data Availability

The data that support the findings of this study are available from the corresponding author upon reasonable request.
