# Supplementary material for: Differential persistence of neutralizing antibody against SARS-CoV-2 in post immunized Bangladeshi population
Source: Sci Rep. 2022 Aug 29;12:14681. doi: 10.1038/s41598-022-18302-9 (PMC9421641; doi:10.1038/s41598-022-18302-9)
Supplement: Supplementary file 1 — Supplementary Tables. [file 41598_2022_18302_MOESM1_ESM.docx]

**Supplementary Information**

**Supplement Table 1: Multivariable analyses of NAb titer after 30 days and 180 days of 2^nd^ dosage vaccination for COVID19 negative subjects (n=402).**

| **Characteristics** | **Nab titers after 30 days** | | **Nab titers after 180 days** | |
| --- | --- | --- | --- | --- |
|  | **β (95% CI)** | **P-value** | **β (95% CI)** | **P-value** |
| **Age** |  |  |  |  |
| <50 years | na |  | na |  |
| ≥50 years | -7.98 (-13.12 – -2.84) | 0.002 | -7.52 (-13.40 – -1.64) | 0.012 |
| **Sex** |  |  |  |  |
| Male | na |  | na |  |
| Female | -2.78 (-7.54 – 1.98) | 0.252 | -2.98 (-8.43 – 2.46) | 0.283 |
| **BMI≥30** | 10.60 (3.40 – 17.79) | 0.004 | 4.38 (-3.84 – 12.60) | 0.296 |
| **Profession** |  |  |  |  |
| Non-medical staff | na |  | na |  |
| Medical staff | -4.37 (-8.76 – -0.24) | 0.051 | -2.31 (-7.33 – 2.71) | 0.367 |
| **Tobacco user** | -8.20 (-14.30 – -2.10) | 0.008 | -14.56 (-21.54 – -7.59) | <0.001 |
| **Comorbidities** |  |  |  |  |
| Diabetes | 10.69 (3.16 –18.22) | 0.005 | 3.16 (-5.45 –11.76) | 0.472 |
| Hypertension | 0.57 (-4.44 – 5.58) | 0.422 | -5.24 (-10.97 – 0.49) | 0.073 |
| Asthma | 1.41 (-3.90 – 6.71) | 0.755 | -18.46 (-24.53 – -12.40) | <0.001 |

**Supplement Table 2: Multivariable analyses of NAb titer after 30 days and 180 days of 2^nd^ dosage vaccination for COVID19 positive subjects (n=129).**

| **Characteristics** | **Nab titers after 30 days** | | **Nab titers after 180 days** | |
| --- | --- | --- | --- | --- |
|  | **β (95% CI)** | **P-value** | **β (95% CI)** | **P-value** |
| **Age** |  |  |  |  |
| <50 years | na |  | na |  |
| ≥50 years | -.65 (-10.71 – 9.40) | 0.899 | 2.58 (-11.15 – 16.31) | 0.713 |
| **Sex** |  |  |  |  |
| Male | na |  | na |  |
| Female | -.65 (-7.62 – 6.31) | 0.854 | -16.27 (-25.78 – -6.76) | 0.001 |
| **BMI≥30** | 10.73 (-4.02 – 25.48) | 0.154 | 14.07 (-6.06 – 34.21) | 0.171 |
| **Profession** |  |  |  |  |
| Non-medical staff | na |  | na |  |
| Medical staff | -2.39 (-9.10 – 4.32) | 0.486 | 1.54 (-7.61 – 10.70) | 0.741 |
| **Tobacco user** | -11.72 (-20.51 – -2.92) | 0.009 | -15.57 (-27.57 – -3.56) | 0.011 |
| **Comorbidities** |  |  |  |  |
| Diabetes | 3.90 (-6.48 – 14.25) | 0.462 | -20.62 (-34.80 – -6.44) | 0.004 |
| Hypertension | 7.07 (-1.81 – 15.94) | 0.119 | 2.57 (-9.54 – 14.69) | 0.677 |
| Asthma | -3.38 (-13.28 – 6.52) | 0.503 | -23.01 (-36.53 – -9.49) | 0.001 |
